# Updated Understanding of Endocrine-Disrupting Substances Involved in the Obesity Epidemic and Their Associated Etiopathogenetic Mechanisms

**DOI:** 10.3390/biomedicines14071455

**Published:** 2026-06-26

**Authors:** Codruța Claudia Gherman Lencu, Cezara Andreea Gerdanovics, Mirela Georgiana Perne, Mircea Vasile Milaciu, Cristian Mureșanu, Geanina Maria Bud, Alexandru Gerdanovics, Teodora Gabriela Alexescu

**Affiliations:** 1 Department of Endocrinology-Fundamental Medicine, Iuliu Hațieganu University of Medicine and Pharmacy, Victor Babeș Street No. 8, 400347 Cluj-Napoca, Romania; 2 4th Department of Internal Medicine, 4th Medical Discipline, Faculty of Medicine, Iuliu Hatieganu University of Medicine and Pharmacy, Republicii Street No. 18, 400015 Cluj-Napoca, Romaniateodora.alexescu@umfcluj.ro (T.G.A.); 3 Laboratory of Molecular and Biomolecular Complexes, Department of Molecular and Isotopic Technologies, National Institute for Research and Development of Isotopic and Molecular Technologies, Donat Street No. 67-103, 400293 Cluj-Napoca, Romania; cristim23@gmail.com; 4 Department of Pneumonology, Faculty of Medicine, Iuliu Hatieganu University of Medicine and Pharmacy, Hașdeu Street No. 6, 400322 Cluj-Napoca, Romania; 5 Neuroscience Department, Iuliu Hatieganu University of Medicine and Pharmacy, 400012 Cluj-Napoca, Romania; 6 Clinical Rehabilitation Hospital, Viilor Street No. 46-50, 400066 Cluj-Napoca, Romania

**Keywords:** obesogens, oxidative stress, epigenetic, microbiota, neuroendocrine, endocrine disruptors

## Abstract

**Purpose:** Obesity is a chronic multifactorial disease whose increasing prevalence cannot be fully explained by excessive caloric intake and sedentary behaviour alone. This review aimed to synthesize current evidence on the role of endocrine-disrupting chemicals (EDCs), particularly obesogenic EDCs, as potential environmental contributors to obesity-related phenotypes, with emphasis on their main classes, etiopathogenetic mechanisms and clinical implications. **Methods:** A structured literature analysis was conducted using PubMed, Web of Science and additional relevant scientific reports and governmental publications. Eligible sources included original research articles, systematic reviews, meta-analyses and authoritative reports addressing endocrine disruption, obesogens, obesity, metabolic dysfunction and related molecular mechanisms. **Results:** The review identified several major classes of obesogenic EDCs, including organotins, bisphenols, phthalates and persistent organic pollutants. These compounds have been linked to obesity-related phenotypes through overlapping mechanisms, including disruption of adipogenesis via estrogen receptor-dependent and independent pathways, PPARγ/RXR activation, altered adipokine signalling, neuroendocrine dysregulation across developmental stages, oxidative stress and pro-inflammatory activation, genetic and epigenetic alterations, gut microbiota-mediated effects and impaired thermoregulation through brown and beige adipose tissue dysfunction. EDC-associated obesity may contribute to metabolic, endocrine, cardiovascular, hepatic and reproductive complications. **Conclusion:** Obesogenic EDCs should be regarded as environmental contributors to obesity that act through interconnected molecular, cellular and systemic pathways. Their biological effects support the need for further mechanistic and epidemiological research, preventive strategies, public education and regulatory measures aimed at reducing exposure.

## 1. Introduction

Obesity is a chronic, relapsing and multifactorial disease characterized by excessive or dysfunctional adiposity, with adverse effects on multiple organs and systems. Beyond its role as a major cardiometabolic risk factor, obesity is associated with endocrine dysregulation, altered hypothalamic–pituitary signalling, insulin resistance, type 2 diabetes, cardiovascular disease, hormone-dependent cancers and mental health disorders [1].

Although excessive caloric intake and sedentary behaviour remain central contributors to the global obesity epidemic, these factors alone do not fully explain its increasing prevalence, early-life onset and marked interindividual variability. In this context, growing attention has been directed toward environmental contributors, particularly endocrine-disrupting chemicals (EDCs), which may interfere with hormonal signalling, metabolic homeostasis and developmental programming.

EDCs comprise a heterogeneous group of exogenous substances capable of altering endocrine function. The total number of known or suspected EDCs has been estimated at approximately 2000–3000 compounds, although only a subset has been sufficiently characterized in terms of endocrine or metabolic effects. Recent expert assessments suggest that approximately 800 compounds are confirmed or strongly suspected EDCs [2], while many others have incomplete toxicological characterization. Among these, a smaller group has been specifically associated with metabolic disruption and obesity-related phenotypes.

This metabolically relevant subgroup, commonly referred to as obesogens, has been implicated in the development and progression of obesity through several converging mechanisms, including altered adipogenesis, impaired metabolic regulation, glucose intolerance, insulin resistance and pancreatic β-cell dysfunction. Their biological effects involve nuclear receptor modulation, hormonal interference, mitochondrial dysfunction, oxidative stress, inflammatory activation, epigenetic changes, gut microbiota alterations and disruption of thermogenic pathways [3]. Therefore, this review aims to synthesize current evidence regarding the main classes of obesogenic EDCs, their proposed etiopathogenetic mechanisms and their clinical implications in obesity and related metabolic disorders. Particular attention is given to distinguishing mechanistic evidence derived from experimental models from associative findings reported in human studies, in order to avoid overinterpretation of causality where the available evidence remains indirect or heterogeneous.

## 2. Methods

This article was designed as a narrative review based on a structured literature search. The aim was to synthesize current evidence regarding endocrine-disrupting chemicals with obesogenic potential, with particular emphasis on their main chemical classes, mechanistic pathways and clinical implications in obesity and obesity-related metabolic dysfunction.

A literature search was conducted in PubMed/MEDLINE and Web of Science, which were selected because of their broad coverage of biomedical, toxicological, endocrinological and multidisciplinary research relevant to endocrine disruption and obesity. Additional relevant information was identified from authoritative reports and scientific documents issued by international and governmental organizations, including the World Health Organization, the Organisation for Economic Co-operation and Development, and regulatory or public health agencies. Additional relevant information was identified from authoritative reports and scientific documents issued by international and governmental organizations, including the World Health Organization, the Organisation for Economic Co-operation and Development and regulatory or public health agencies. The search covered publications available from January 2010 to November 2025, with additional seminal earlier studies included when they were considered essential for mechanistic or conceptual background. The search covered publications available from January 2010 to November 2025, with additional seminal earlier studies included when they were considered essential for mechanistic or conceptual background. The final database search was performed on 22 November 2025.

The search strategy combined terms related to endocrine disruption, obesogenic chemicals, obesity, metabolic dysfunction, and mechanistic pathways. The following terms and combinations were used: “endocrine-disrupting chemicals” OR “endocrine disruptors” OR “EDCs”; “obesogens” OR “obesogenic chemicals”; “obesity” OR “adipogenesis” OR “adipocyte differentiation” OR “metabolic syndrome” OR “insulin resistance”; “bisphenol A” OR “bisphenols” OR “phthalates” OR “organotins” OR “tributyltin” OR “persistent organic pollutants” OR “dioxins” OR “polychlorinated biphenyls”; and “oxidative stress” OR “inflammation” OR “epigenetics” OR “gut microbiota” OR “neuroendocrine” OR “thermogenesis” OR “brown adipose tissue”. Boolean operators were adapted according to the requirements of each database.

Eligible sources included original experimental studies, observational human studies, systematic reviews, meta-analyses, narrative reviews with high relevance to the topic, mechanistic studies and authoritative scientific reports addressing endocrine disruption, obesogenic effects, obesity-related metabolic outcomes or molecular pathways relevant to adipogenesis, neuroendocrine regulation, oxidative stress, inflammation, epigenetic regulation, gut microbiota and thermoregulation. Studies were excluded if they were not available in English, were not directly related to endocrine disruption or obesity-related outcomes, focused exclusively on unrelated toxicological endpoints or lacked sufficient mechanistic or clinical relevance to the scope of the review.

The study selection process was performed in two stages. After removal of duplicate records, titles and abstracts were screened for relevance to endocrine disruption, obesogenic mechanisms and obesity-related metabolic outcomes. Potentially eligible articles were subsequently assessed in full text to determine whether they contributed to one or more of the predefined thematic domains of the review. These domains included adipogenesis, neuroendocrine regulation, oxidative stress and inflammation, genetic and epigenetic mechanisms, gut microbiota, thermoregulation, exposure assessment and clinical implications.

Study selection was performed by two authors, and uncertainties regarding eligibility or thematic relevance were resolved through discussion and consensus. Priority was given to recent publications, studies with clear mechanistic or clinical relevance, systematic reviews and meta-analyses, and authoritative reports that helped distinguish experimental evidence from human epidemiological findings. Reference lists of highly relevant reviews and included articles were also examined to identify additional publications that might not have been captured by the database search.

Evidence was synthesized narratively and organized according to the major classes of obesogenic endocrine-disrupting chemicals, principal mechanisms of action and level of supporting evidence. Because this manuscript was designed as a narrative review rather than a systematic review or meta-analysis, no formal PRISMA flow diagram, risk-of-bias assessment or quantitative evidence grading was performed.

For interpretation, the included evidence was categorized according to study type and inferential strength. In vitro and cell culture studies were considered primarily mechanistic and were used to describe receptor-level, cellular, mitochondrial, inflammatory, adipogenic, epigenetic and microbiota-related pathways. Animal studies were interpreted as supporting biological plausibility and developmental or dose-related effects, particularly when they involved controlled exposure models. Human observational studies were considered relevant for associations between EDC exposure biomarkers and obesity-related phenotypes but were not interpreted as definitive evidence of causality because of potential confounding, reverse causation, co-exposure to chemical mixtures, variability in exposure assessment, and heterogeneity of metabolic outcomes. Systematic reviews, meta-analyses, and expert or regulatory reports were used to contextualize the consistency, limitations, and public health relevance of the evidence. Accordingly, conclusions were framed according to the strength of the available evidence, distinguishing robust mechanistic support from associative human findings and from hypotheses that remain speculative.

## 3. The Epidemic of Obesity

Obesity has become a major global public health challenge, affecting individuals across age groups, sex, and socioeconomic categories. Its rising prevalence reflects the interaction of multiple biological, behavioural, social, and environmental determinants rather than the effect of a single causal pathway. In addition to excessive caloric intake and reduced physical activity, several contextual factors contribute to obesity risk, including food marketing, limited access to affordable healthy foods, urbanization, psychological stress, sleep deprivation, and weight-promoting medications [4,5].

The clinical burden of obesity is substantial, as it increases the risk of type 2 diabetes, cardiovascular disease, stroke, several cancers, reduced quality of life, and mental health disorders. These consequences generate major costs for healthcare systems and societies. Therefore, obesity should be approached as a complex chronic disease requiring coordinated prevention and intervention strategies that address not only individual behaviour, but also environmental and biological contributors.

In this broader framework, endocrine-disrupting chemicals represent a relevant environmental factor that may contribute to obesity susceptibility. Their effects may begin during early developmental windows and persist across the life course through endocrine, metabolic, inflammatory, epigenetic, and neuroendocrine mechanisms. This perspective supports the need to examine obesogenic EDCs as contributors to the current obesity epidemic, alongside established dietary, behavioural, and socioeconomic drivers.

## 4. Main Classes of Obesogenic EDCs and Their Actions Related to Obesity

Several classes of endocrine-disrupting chemicals have been linked to obesity-related phenotypes, including organotins, bisphenols, phthalates and persistent organic pollutants. The strength of evidence differs across these classes and outcomes. For some compounds, such as organotins, the obesogenic effect is supported mainly by experimental and mechanistic studies involving nuclear receptor activation and adipocyte differentiation. For bisphenols, phthalates and persistent organic pollutants, the evidence includes both experimental data and human observational associations, although the latter do not establish causality. Although these compounds differ in chemical structure, sources of exposure and persistence, their proposed biological actions may converge on adipogenesis, nuclear receptor signalling, lipid metabolism, insulin sensitivity, mitochondrial function, thyroid hormone regulation and inflammatory pathways. Organotins are found in marine ecosystems and in several consumer or industrial products, including polyvinyl chloride (PVC)-containing materials, flooring, pipes, toys, and food packaging. They may contaminate food through packaging, municipal waste, and water and have also been detected in house dust and aquatic organisms. Among them, tributyltin (TBT) is one of the best-characterized obesogens. TBT activates peroxisome proliferator-activated receptor gamma (PPARγ) and retinoid X receptor (RXR), two nuclear receptors involved in adipocyte differentiation and lipid storage [6]. Through these pathways, TBT may promote the differentiation of mesenchymal stromal cells and preadipocytes into mature adipocytes, thereby increasing adipose tissue expansion and fat accumulation.

Bisphenols are widely used in consumer products, including polycarbonate plastics, water bottles, food containers, bottle tops, and epoxy resins used in the lining of metal cans and water supply pipes. Bisphenol A (BPA), bisphenol AF (BPAF), and bisphenol C (BPC) can interact with estrogen receptors, acting as agonists or antagonists depending on the receptor subtype and tissue context [7]. In addition, polyhalogenated bisphenols may interfere with PPARγ-regulated adipogenesis and metabolic function. These compounds have been associated with altered lipid metabolism-related gene expression, pancreatic β-cell dysfunction, insulin resistance, and obesity-related metabolic disturbances.

**Figure 1:**
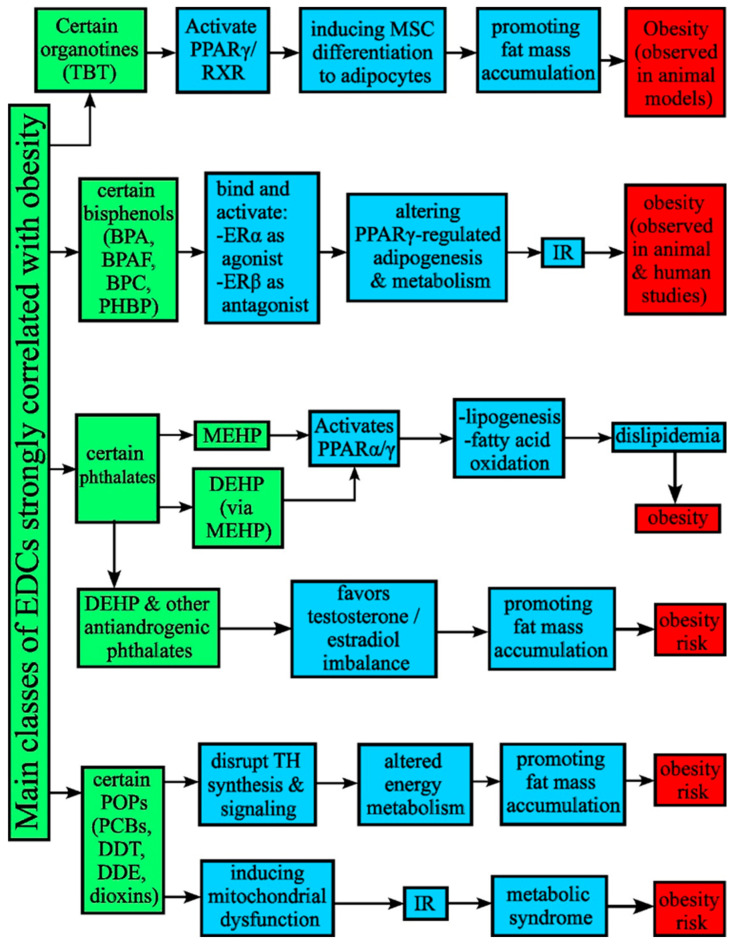
Overview of the major obesogenic endocrine-disrupting chemicals and their biological effects. This schematic representation summarizes four major classes of EDCs associated with obesity and highlights the key molecular and metabolic pathways involved in obesogenic outcomes.

Phthalates are commonly used as plasticizers in PVC products to increase flexibility and are also present in adhesives, flooring materials, cosmetics, electronic devices, and other consumer products. Certain phthalate metabolites, such as mono-(2-ethylhexyl) phthalate (MEHP), may activate PPARα and PPARγ, thereby influencing lipid metabolism, adipocyte function, and reproductive endocrine pathways [8]. Di-2-ethylhexyl phthalate (DEHP) has also been shown to promote lipogenesis and alter fatty acid oxidation (Figure 1), disturbing the balance between lipid synthesis and catabolism. These effects may contribute to triglyceride accumulation, dyslipidemia, insulin resistance, and increased obesity risk, particularly in experimental models [9].

Phthalates, including DEHP and related compounds, may also exert anti-androgenic effects, thereby altering the balance between oestrogenic and androgenic signalling. This endocrine imbalance may be relevant not only for reproductive outcomes, but also for adipose tissue distribution and metabolic regulation. In men, estradiol appears to play an important role in the regulation of adiposity, and even short-term estradiol deprivation has been associated with increased fat mass accumulation [10].

Persistent organic pollutants (POPs), including polychlorinated biphenyls (PCBs), dichlorodiphenyltrichloroethane (DDT) and its metabolite 1,1-dichloro-2,2-bis(p-chlorophenyl)ethylene (DDE), as well as dioxins, are lipophilic environmental contaminants that persist in the environment and accumulate in adipose tissue [11]. They may be found in air, water, soil, legacy pesticides, industrial chemicals, and incineration-related waste products. Human exposure occurs mainly through contaminated food, particularly animal-derived products and fish, but also through contaminated air and consumer products.

POPs have been associated with obesity-related metabolic dysfunction through several mechanisms, including disruption of thyroid hormone homeostasis, impaired energy expenditure, altered lipid metabolism, mitochondrial dysfunction, oxidative stress, and insulin resistance [12,13]. Because thyroid hormones are essential regulators of basal metabolic rate, POP-induced interference with thyroid function may contribute to reduced energy expenditure and fat accumulation. In addition, chronic low-dose exposure to POPs may impair mitochondrial oxidative phosphorylation, thereby promoting insulin resistance and metabolic syndrome, conditions closely associated with overweight and obesity [13].

## 5. Exposure Assessment, Environmentally Relevant Doses, Critical Windows and Mixture Effects

A major challenge in evaluating the obesogenic relevance of endocrine-disrupting chemicals is the accurate assessment of exposure. Human exposure may occur through food, water, air, dust, packaging materials, plastics, cosmetics and other consumer products, depending on the chemical class and persistence of the compound [2,14]. Exposure assessment can include environmental monitoring, dietary or occupational exposure evaluation, and biomonitoring approaches using urine, blood, serum, plasma or lipid-rich tissues. For non-persistent chemicals such as bisphenols and phthalates, urinary metabolites are commonly used as exposure biomarkers, but they often reflect recent exposure and may vary over time [8,14]. In contrast, persistent organic pollutants are lipophilic compounds that can accumulate in adipose tissue and may be detected over longer periods, although interpretation may be influenced by lipid storage, weight change and mobilization from adipose compartments [11].

The distinction between experimental exposure levels and real-world human exposure is particularly important. Mechanistic studies often use controlled exposure conditions that are useful for identifying biological pathways, but these may not fully reproduce chronic low-level exposure in humans. Conversely, real-world exposure usually involves repeated contact with multiple low-dose chemicals rather than isolated exposure to a single compound. This is relevant for obesogenic EDCs because endocrine and metabolic effects may depend on route, dose, duration, timing, persistence, metabolism and tissue distribution [14,15]. Low-dose exposure is particularly important for persistent pollutants, as human and in vitro evidence suggests that low-dose POP exposure may affect mitochondrial function and metabolic regulation [13].

Critical windows of susceptibility are also central to obesogenic EDC research. Exposure during preconception, gametogenesis, pregnancy, fetal development, infancy, childhood, puberty and adolescence may have different biological consequences because endocrine, neuroendocrine, adipose, hepatic, pancreatic, immune and epigenetic systems are dynamically regulated during these periods [15,16]. Early-life exposure may influence developmental programming of adipogenesis, appetite regulation, insulin sensitivity, thyroid hormone signalling, mitochondrial function and epigenetic regulation. However, the degree to which these mechanisms explain human obesity remains incompletely established and should be interpreted cautiously.

Low-dose exposure and dose–response complexity further complicate interpretation. Unlike classical toxicology models, endocrine disruption may involve biological effects at exposure levels that are difficult to extrapolate directly from high-dose experimental models. This issue is particularly relevant when chemicals interact with hormone receptors, endocrine feedback loops, mitochondrial function or metabolic signalling pathways [13,14,15]. Therefore, experimental findings should be interpreted in relation to environmentally relevant concentrations, exposure route, timing, duration and the biological context in which exposure occurs.

Real-life exposure also occurs as mixtures rather than as isolated compounds. Individuals may be simultaneously exposed to bisphenols, phthalates, organotins, persistent organic pollutants and other environmental chemicals through dietary, household, occupational and environmental sources [2,6,8,14]. These compounds may converge on shared pathways, including PPARγ/RXR signalling, thyroid hormone disruption, oxidative stress, mitochondrial dysfunction, inflammation, gut microbiota alterations and epigenetic regulation [13,15]. Therefore, evaluating one compound at a time may underestimate or misrepresent the biological relevance of combined exposures. Future studies should prioritize repeated exposure assessment, environmentally relevant dose ranges, mixture-based models, longitudinal human cohorts and standardized metabolic outcomes. Mixture toxicology represents a major methodological challenge because combined exposures may not simply reflect the sum of individual chemical effects. Chemicals within a mixture may act additively when they influence similar biological targets, synergistically when one compound amplifies the effect of another, or antagonistically when one exposure attenuates the biological effect of another. In the context of obesogenic EDCs, this is particularly relevant because different chemical classes may converge on overlapping endocrine and metabolic pathways, including PPARγ/RXR activation, thyroid hormone disruption, mitochondrial dysfunction, oxidative stress, inflammatory signalling, adipokine dysregulation, gut microbiota alterations and epigenetic regulation. Consequently, studying one compound at a time may not adequately capture the biological complexity of real-world exposure.

Cumulative exposure assessment is also difficult because EDCs differ in persistence, metabolism, biological half-life, tissue distribution and timing of exposure. Non-persistent compounds, such as bisphenols and phthalates, may show substantial short-term variability and require repeated biomonitoring to better estimate usual exposure, whereas persistent organic pollutants may accumulate in lipid-rich tissues and reflect longer-term exposure. Moreover, exposure biomarkers are often correlated because individuals are exposed to multiple chemicals through shared sources, such as diet, food packaging, indoor dust, plastics, personal care products and occupational environments. These correlations make it difficult to isolate the effect of a single compound and may lead to exposure misclassification or residual confounding in epidemiological studies. Future research should therefore use mixture-based analytical approaches, repeated exposure measurements, longitudinal designs and standardized obesity-related outcomes to better evaluate the cumulative metabolic relevance of EDC exposure.

## 6. Detailed Presentation of the Mechanisms Through Which EDCs May Contribute to Obesity-Related Phenotypes

After outlining the main classes of obesogenic endocrine-disrupting chemicals, it is important to examine the molecular and cellular mechanisms through which these compounds may contribute to obesity. Although individual EDCs differ in their chemical structure, persistence, exposure sources and receptor affinity, many of their biological effects converge on common endocrine and metabolic pathways. Understanding these shared mechanisms provides a framework for interpreting substance-specific findings and for integrating evidence across experimental, epidemiological and translational studies.

Obesogens are environmental chemicals that may contribute to obesity-related phenotypes by altering metabolic regulation, disrupting appetite and satiety pathways, impairing lipid homeostasis, increasing adipocyte hypertrophy, or stimulating adipogenic differentiation and adipocyte hyperplasia during critical developmental windows and later in life [16]. For clarity, the mechanisms involved in obesogenic EDC action can be grouped into six major categories: adipogenesis disruption; neuroendocrine dysregulation; oxidative stress and pro-inflammatory activation; genetic and epigenetic alterations; gut microbiota-mediated effects; and impaired thermoregulation.

These pathways are not mutually exclusive. According to the Endocrine Society Scientific Statement, EDCs may act through multiple mechanisms, including estrogenic, antiandrogenic and thyroid-related pathways; modulation of PPARγ and retinoid signalling; interactions with nuclear receptors, steroidogenic enzymes, neurotransmitter receptors and other endocrine or metabolic targets [15]. Therefore, obesogenic effects should be interpreted as the result of overlapping and interacting mechanisms rather than isolated linear pathways. The mechanisms discussed below should therefore be interpreted according to the type of evidence supporting them. In vitro and animal studies provide important mechanistic plausibility, especially for receptor activation, adipocyte differentiation, mitochondrial dysfunction, oxidative stress and inflammatory signalling. However, translation to humans remains less certain because real-life exposure occurs through complex mixtures, at variable doses and during different windows of susceptibility. Human studies are valuable for identifying associations between exposure biomarkers and obesity-related phenotypes, but they are frequently limited by cross-sectional design, residual confounding, reverse causation and heterogeneous exposure assessment.

To avoid overinterpretation, the mechanisms described in the following sections should not be interpreted as complete causal pathways fully demonstrated in humans. Some mechanisms, such as PPARγ/RXR activation, adipocyte differentiation, mitochondrial dysfunction, oxidative stress and inflammatory signalling, are supported predominantly by in vitro and animal evidence. Human studies mainly provide associations between exposure biomarkers and obesity-related phenotypes, insulin resistance or metabolic dysfunction. Other mechanisms, including long-term neuroendocrine programming, transgenerational transmission, gut–brain–microbiota interactions and brown or beige adipose tissue dysfunction, remain biologically plausible but incompletely validated in humans. Therefore, each pathway is presented according to its current evidentiary support rather than as a definitive sequence from exposure to human obesity.

### 6.1. Adipogenesis Disruption Mechanism by Obesogenic EDCs

Adipogenesis is the process through which mesenchymal stromal cells differentiate into preadipocytes and subsequently into mature adipocytes capable of lipid storage and endocrine activity [17]. Although adipocyte number is largely established during childhood and adolescence, adipose tissue remains dynamic throughout life, with changes in adipocyte size, turnover and metabolic function contributing to body fat accumulation and obesity-related complications [18].

**Figure 2:**
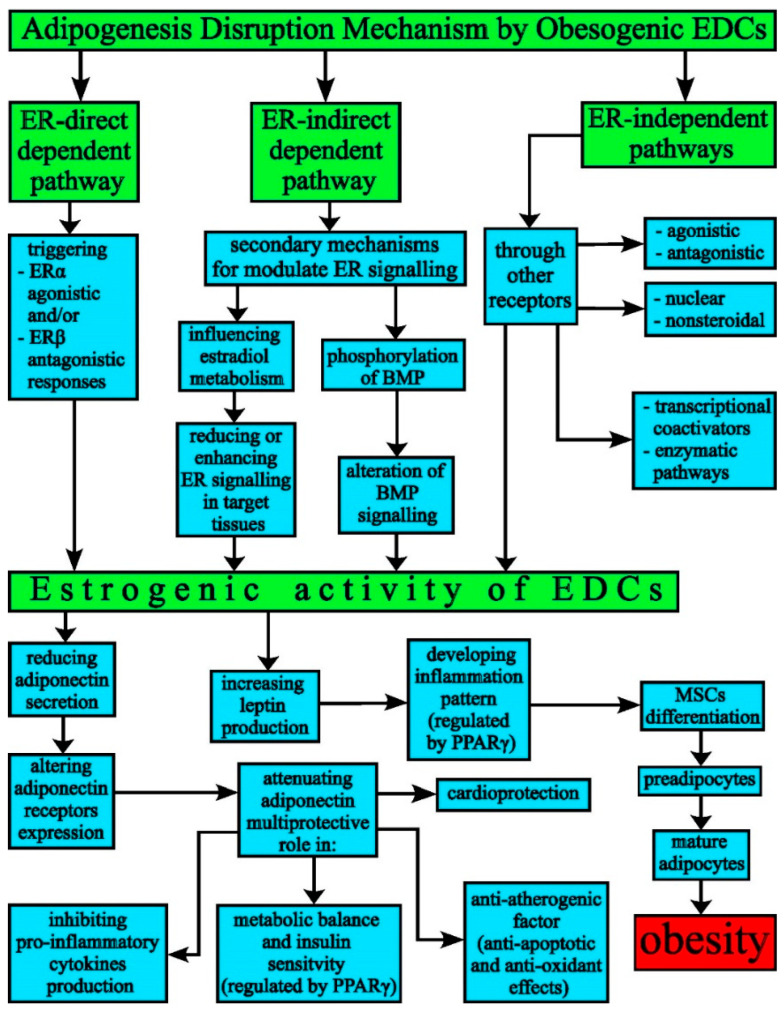
Adipogenesis disruption induced by obesogenic endocrine-disrupting chemicals. The figure illustrates how obesogenic EDCs may interfere with adipocyte differentiation and adipose tissue homeostasis through ER-dependent, ER-indirect and ER-independent pathways. These mechanisms may involve altered estrogen signalling PPARγ/RXR activation, adipokine dysregulation, impaired insulin sensitivity and increased differentiation of mesenchymal stromal cells and preadipocytes into mature lipid-storing adipocytes.

Obesogenic EDCs may interfere with adipogenesis through estrogen receptor (ER)-dependent and ER-independent mechanisms, as illustrated in Figure 2. In ER-dependent pathways, EDCs may bind directly to estrogen receptors and act as agonists or antagonists, depending on the compound, receptor subtype, tissue context and exposure level. In ER-indirect pathways, EDCs may alter estrogen signalling without directly binding to ERs, for example by modifying estradiol metabolism, receptor phosphorylation or bone morphogenetic protein (BMP)-related signalling. Bisphenol A (BPA) is a relevant example, as it can behave as a xenoestrogen with lower affinity for classical genomic ERs than estradiol, while still reaching biologically active concentrations in exposed individuals [7,14].

In ER-independent pathways, obesogenic EDCs may act through other molecular targets, including nuclear receptors, transcriptional co-regulators, steroidogenic enzymes and nonsteroidal receptor systems. These interactions may influence adipocyte differentiation, lipid accumulation, insulin sensitivity and adipokine secretion. In particular, EDC-related modulation of adiponectin is relevant because adiponectin contributes to insulin sensitivity, anti-inflammatory signalling, anti-atherogenic protection and cardiometabolic homeostasis [19]. Reduced adiponectin secretion, especially when accompanied by increased leptin production or leptin resistance, may promote a pro-inflammatory and metabolically dysfunctional adipose tissue phenotype.

A central mechanism linking EDC exposure to adipogenesis involves the activation of peroxisome proliferator-activated receptor gamma (PPARγ), a nuclear receptor with a key physiological role in adipocyte differentiation, lipid storage, insulin sensitivity and metabolic regulation [20]. Certain obesogenic EDCs, including organotins and some phthalate metabolites, may aberrantly activate PPARγ and/or retinoid X receptor (RXR), thereby favouring the commitment of mesenchymal stromal cells and preadipocytes toward the adipocyte lineage. This may increase the formation of mature lipid-storing adipocytes and contribute to adipose tissue expansion.

Overall, obesogenic EDCs may disrupt adipose tissue homeostasis by combining ER-mediated effects, PPARγ/RXR activation, altered adipokine secretion and impaired insulin signalling. These mechanisms support the concept that environmental chemicals may contribute not only to increased fat accumulation but also to the development of metabolically dysfunctional adipose tissue.

Once the levels of this hormone are altered, its multiprotective role is attenuated, because adiponectin is a polypeptide hormone secreted by adipocytes and it has a protecting role and regulation in inhibiting pro-inflammatory cytokines production, and it also acts as an anti-atherogenic factor, having anti-apoptotic and anti-oxidant effects, metabolic balance and cardioprotection, including insulin sensitivity [19].

If adiponectin expression/secretion from adipocytes is reduced, and/or consequently leptin production is increased then the result is a pattern that promotes inflammation. PPARs are a family of nuclear receptors, also known as transcription factors activated by fatty acids, which regulate energy homeostasis, metabolic function, lipid metabolism, inflammation, cellular growth, and differentiation [20]. Several studies have demonstrated a direct correlation between obesity, waist circumference, and urinary BPA concentrations in Korean adults.

In summary, experimental evidence suggests that selected obesogenic EDCs may interfere with adipogenesis through PPARγ activation, favouring the differentiation of MSCs and preadipocytes into mature adipocytes. Because PPARγ plays a central role in adipogenesis and insulin sensitivity, these mechanisms support the biological plausibility of a link between EDC exposure and adipose tissue dysfunction.

### 6.2. Proposed Neuroendocrine Mechanisms of Obesogenic EDCs

**Figure 3:**
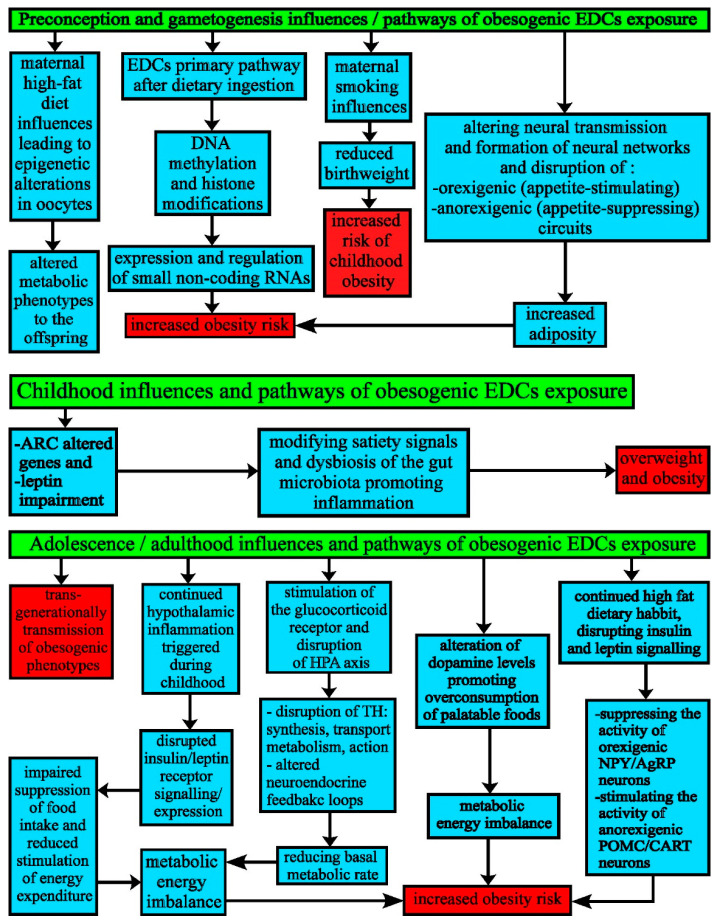
Neuroendocrine disruption induced by obesogenic EDCs across the life course.

Experimental and epidemiological evidence suggests that exposure to certain endocrine disruptors, both in utero and later in life, may interfere with hypothalamic pathways involved in appetite and energy homeostasis. These proposed effects may involve orexigenic and anorexigenic neuropeptide systems within the arcuate nucleus (ARC), although the complete pathway linking EDC exposure, hypothalamic dysfunction and human obesity remains incompletely demonstrated. To describe the neuroendocrine mechanism as comprehensively as possible, it is necessary to understand how exposure to obesogenic EDCs manifests across developmental stages, beginning with gametogenesis and embryogenesis, and continuing through the perinatal period, childhood, and adulthood, as illustrated in Figure 3.

#### 6.2.1. Neuroendocrine Mechanisms of Obseogenic EDCs During Preconception and Gametogenesis

During preconception and gametogenesis, maternal preconceptional consumption of a high-fat diet is sufficient to induce epigenetic modifications in oocytes, thereby transmitting altered metabolic phenotypes to the offspring [21].

Considering the presence of obesogenic EDCs in food, water, and air, these substances may alter epigenetic marks, including sperm DNA methylation, histone modifications, and the expression and regulation of small non-coding RNAs, because paternal exposure to EDCs has also been associated with an increased predisposition to disease, infertility, testicular disorders, obesity, and polycystic ovary syndrome in females, mediated via epigenetic alterations during gametogenesis [22]. For instance, maternal smoking during pregnancy can result in reduced birthweight and an increased risk of developing obesity during childhood. We highlight that exposure to EDCs can directly alter the epigenetic modifications of germline cells, leading to changes in epigenetic information and associated phenotypes.

Continued exposure to EDCs allows these substances to cross the placental barrier via umbilical cord blood, reaching fetal circulation and accumulating in fetal tissues, thereby impairing placental development, growth, and function. Consequently, the embryo or fetus is exposed during critical windows of neural patterning. Subsequently, EDCs can cross the blood–brain barrier, potentially inducing hypothalamic inflammation and disrupting the reproductive axis [23].

#### 6.2.2. Neuroendocrine Mechanisms of Obseogenic EDCs During Childhood

During childhood, experimental evidence suggests that continued exposure to certain obesogenic EDCs may alter hypothalamic arcuate nucleus (ARC) pathways involved in reproduction and energy homeostasis, although the extent to which these mechanisms translate directly to human childhood obesity remains incompletely established. Figure 3 illustrate a simplified more comprehensible version of the main pathways and influences associated with exposure to obesogenic EDCs, as discussed in Chapter 4. The figure highlights risk factors beginning in the preconception period—such as a high-fat diet and smoking—followed by cumulative effects of continuous EDC exposure during pregnancy. These exposures exert lasting impacts on health throughout subsequent life stages, including childhood, adolescence, and adulthood, during which additional complications may arise or existing ones may be exacerbated. Ultimately, these mechanisms contribute to the development of obesity and related diseases, with potential intergenerational or transgenerational effects suggested mainly by experimental and epigenetic evidence but not definitively established as a causal pathway in humans.

Because the ARC contains orexigenic (NPY/AgRP) and anorexigenic (POMC/CART) neurons, it plays a central role in regulating food intake and energy homeostasis by responding to hormonal signals such as leptin and insulin [24]. This activity promotes appetite and diminishes satiety signalling, allowing food consumption to continue for longer periods than normal. EDCs may disrupt the production of hormones, including peptide hormones and steroids, and have also been associated with gut microbiota dysbiosis in experimental and observational studies [25], thereby, modifying satiety signals reaching the hypothalamus.

#### 6.2.3. Neuroendocrine Mechanisms of Obseogenic EDCs During Adolescence and Adulthood

During adolescence and adulthood, neuroendocrine dysfunction may become chronic, continuing to modulate pubertal timing and neural circuits in the hypothalamus, thereby leading to the dysregulation of gonadotrophin hormone (FSH/LH) secretion by the pituitary. Moreover, EDCs can alter hypothalamic regulation of puberty, as exemplified by the fungicide ketoconazole, while others may modify the fate of hormone-producing or hormone-responsive cells [26], or affect reproductive function by influencing the hypothalamic–pituitary–gonadal axis [27].

Early modifications of neural transmission and the formation of neural networks establish a long-term “wiring” bias towards orexigenic (appetite-stimulating) or anorexigenic (appetite-suppressing) circuits, because prenatal exposure to several EDCs has been associated in epidemiological studies with adverse neurobehavioural outcomes and increased adiposity or obesity risk, although causality remains difficult to establish because of confounding, co-exposures and variability in exposure timing. This heightened vulnerability occurs because the fetus, infant, or child exhibits increased sensitivity to environmental stressors, which disrupt hormonally mediated processes critical for growth and development [28]. Moreover, because EDCs are capable of crossing the blood–brain barrier, they may potentially induce hypothalamic inflammation and disrupt the reproductive axis.

Once this occurs, a high-fat diet, which may also contain obesogenic EDCs, can exacerbate hypothalamic inflammation and impair insulin and leptin signalling, further suppressing the activity of orexigenic neuropeptide Y (NPY)/agouti-related protein (AgRP) neurons, while concurrently stimulating anorexigenic pro-opiomelanocortin (POMC)/cocaine- and amphetamine-regulated transcript (CART) neurons [23].

Because hypothalamic leptin signalling is closely involved in body weight regulation, impaired leptin signalling may contribute to altered appetite control, reduced energy expenditure and increased susceptibility to weight gain. However, the contribution of EDC-induced leptin resistance to human obesity remains an area of ongoing investigation [29].

Then, the aforementioned EDC-induced hypothalamic inflammation leads to disrupted receptor signalling or altered receptor expression, thereby increasing leptin and IR, which in turn impairs both the suppression of food intake and the stimulation of energy expenditure. EDCs can stimulate the glucocorticoid receptor, modulate cortisol rhythms, and alter the levels and activity of the hypothalamic–pituitary–adrenal (HPA) axis [30]. They may also affect TH synthesis, transport, metabolism, and action in various ways [31], thereby reducing basal metabolic rate via neuroendocrine feedback loops, as THs are essential regulators of basal metabolic rate.

Another neuroendocrine mechanism of obesogenic EDCs, distinct from those associated with hypothalamic effects, involves the alteration of dopamine levels in the brain, which mediates the reward value of food and promotes overconsumption of palatable foods, beverages, and, potentially, substance abuse [32].

The association with obesity is also mediated through alterations in gut hormonal signalling and changes in the microbiome induced by EDCs, which amplify central orexigenic impulses. EDCs have been shown to induce modifications in the gut microbiota via the gut–brain–microbiota axis, thereby conferring increased susceptibility to obesity and neurodevelopmental disorders [33]. With advancing age, all of the neuroendocrine mechanisms described contribute to the exacerbation of health problems, which may persist throughout adult life and, in some cases, be transmitted transgenerationally, promoting obesogenic phenotypes [23].

We briefly highlight that on childhood, adolescence and adulthood, the primary circuit disrupted by certain EDCs, such as BPA and TBT, involves orexigenic (NPY/AgRP) neurons, which stimulate appetite, and anorexigenic (POMC/MCH) neurons, which suppress appetite, both of which are integrated within the ARC [34]. These proposed disruptions may contribute to increased food intake, diminished satiety, reduced sympathetic tone and decreased energy expenditure, thereby supporting a biologically plausible link with obesity-related phenotypes. However, the complete neuroendocrine cascade remains incompletely validated in humans.

### 6.3. Oxidative Stress and Pro-Inflammatory Pathways Potentially Involved in EDC-Related Metabolic Dysfunction

**Figure 4:**
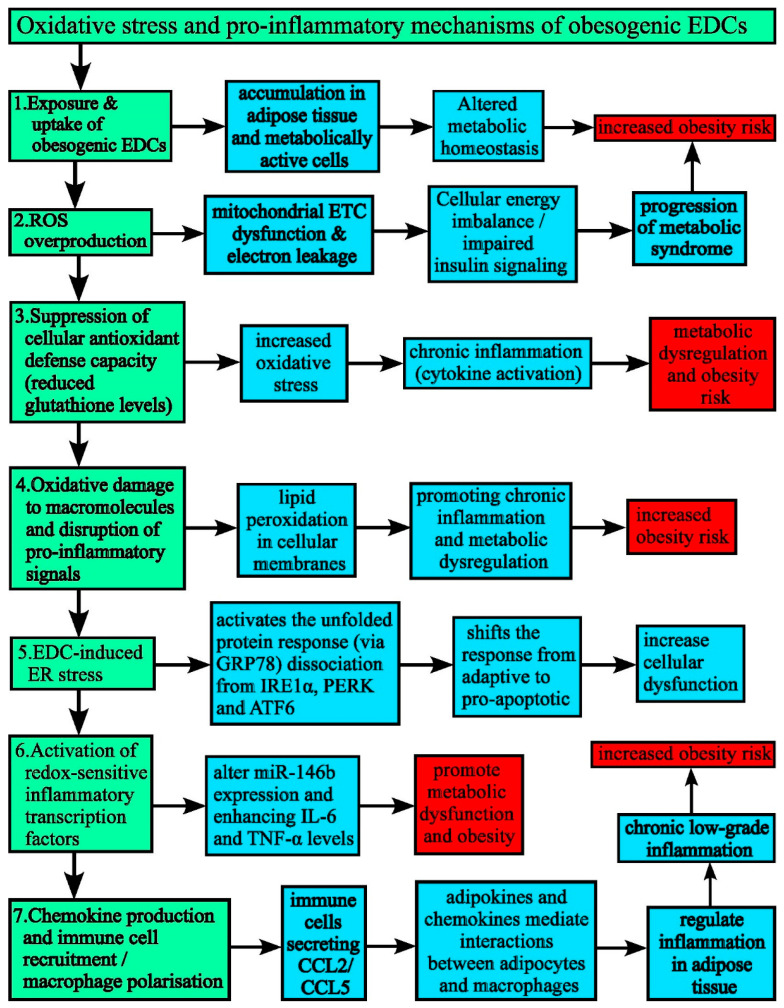
Sequential and overlapping oxidative stress–inflammatory mechanisms induced by obesogenic EDCs.

Oxidative stress and inflammatory mechanisms may occur at one or multiple stages, depending on the chemical class, dose, duration of exposure, persistence, tissue accumulation and individual susceptibility. Although some EDCs can accumulate through repeated exposure via food, water, air or consumer products, downstream oxidative and inflammatory effects should not be considered inevitable. Rather, they represent biologically plausible pathways supported mainly by experimental evidence, with variable degrees of confirmation in human studies. Figure 4 illustrates a proposed sequence of oxidative stress and inflammatory events potentially associated with obesogenic EDC exposure, based mainly on experimental evidence. These processes may overlap or interact across interconnected pathways, but the complete cascade has not been fully demonstrated in humans.

We are analyzing exposure and cellular uptake, the primary induction of reactive oxygen species (ROS), and the impairment of antioxidant defences, followed by oxidative damage to macromolecules and the subsequent generation of inflammatory signals.

Our review also addresses endoplasmic reticulum (ER) stress and the unfolded protein response (UPR), activation of redox-sensitive inflammatory transcription factors, and priming and activation of the NOD-like receptor family pyrin domain containing 3 (NLRP3) inflammasome, all of which are associated with adipokine dysregulation linked to inflammation. Subsequently, it is important to consider chemokine production and the recruitment and polarization of immune cells, including macrophages.

#### 6.3.1. Exposure and Cellular Uptake of Obesogenic EDCs

After exposure, most EDCs may accumulate in adipose tissue adipocytes, affect endothelial and epithelial cells penetrate cell membranes, impacting mitochondria and inducing ER stress [35].

#### 6.3.2. Generation of Reactive Oxygen Species

In experimental models, after cellular internalization, several EDCs have been shown to directly or indirectly increase the production of ROS and reactive nitrogen species causing mitochondrial electron transport chain (ETC) dysfunction and electron leakage. The resulting excessive ROS production ultimately leads to cellular damage [36].

Although ROS overproduction can be harmful, it also plays a role in signalling, and under certain circumstances, their generation may be beneficial by facilitating cellular adaptation to stress.

#### 6.3.3. Impairment of Antioxidant Defences

The antioxidant defence refers to the body’s intrinsic capacity to neutralize harmful molecules, commonly referred to as “free radicals”, which can induce oxidative stress. Oxidative stress arises when oxidant production exceeds the capacity of antioxidant systems to detoxify them, resulting in damage to cells, proteins, and DNA. For example, exposure to electronic cigarette aerosol extracts has been shown to suppress cellular antioxidant defences and induce significant DNA damage [37].

The mechanism underlying the suppression of cellular antioxidant defence capacity involves glucocorticoids, which are activated by elevated levels of 11β-hydroxysteroid dehydrogenase-1 (11β-HSD1) and play a role in energy provision for metabolic demands, potentially leading to impaired nuclear factor (erythroid-derived 2)-like 2 (Nrf2)-dependent antioxidant responses. In such cases, it is postulated that pharmacological inhibition of 11β-HSD1 may enhance the cellular capacity to cope with oxidative stress; however, further in vivo studies are required.

#### 6.3.4. Oxidative Damage to Macromolecules and Generation of Inflammatory Signals

This stage is characterized by persistent, long-term ROS production, which disrupts the balance of pro-inflammatory signalling. Certain EDCs have been shown to induce oxidative stress in human studies, initiating lipid peroxidation in cellular membranes and then lipid peroxidation generates additional ROS, inducing tissue injury through the formation of lipid peroxidation products [38].

Experimental evidence supports the ability of several EDCs to impair mitochondrial function through multiple pathways, including disruption of the electron transport chain, perturbation of Ca^2+^ homeostasis, increased ROS production and activation of mitochondrial apoptotic pathways [36]. These perturbations are further exacerbated by the persistence and continuous accumulation of EDCs in the body, extending their deleterious effects across subsequent stages.

#### 6.3.5. Endoplasmic Reticulum Stress and the Unfolded Protein Response

At this stage, oxidative damage and EDC interactions continue to generate further perturbations. For instance, EDCs can trigger ER stress, which subsequently activates a signalling network known as the unfolded protein response (UPR) to restore ER homeostasis. If prolonged, UPR signalling may initiate cell death pathways when ER homeostasis is not restored over an extended period, because it is well established that ER stress can induce apoptosis [39]. At this stage, ER stress acts synergistically with ROS to amplify downstream inflammatory signalling.

#### 6.3.6. Activation of Redox-Sensitive Inflammatory Transcription Factors

During this stage, ROS, ER stress, and danger-associated molecular patterns (DAMPs) converge to activate a cascade of inflammatory responses via the nuclear factor-kappa B (NF-κB) pathway, a transcription factor that coordinates innate and adaptive immunity, inflammation, and apoptotic cell death [40].

#### 6.3.7. Chemokine Production and Immune Cell Recruitment/Macrophage Polarization

Scientific studies have demonstrated that obesity is associated with immune responses involving chemokines secreted by immune cells, such as Chemokine (C-C motif) Ligand 2 and 5 (CCL2/CCL5), because both adipokines and chemokines mediate interactions between adipocytes and macrophages and regulate inflammation in adipose tissue [41].

It is important to note that any oxidative stress–driven interference with insulin signalling has metabolic consequences relevant to weight gain, while associated epigenetic changes consolidate chronic inflammation.

### 6.4. Genetic and Epigenetic Mechanisms of EDCs

Experimental and epidemiological evidence suggests that EDC exposure may be associated with epigenetic modifications affecting DNA methylation, histone regulation and non-coding RNA expression. These changes may influence immune, reproductive and metabolic pathways, although their long-term causal contribution to human obesity and related diseases remains incompletely established [42].

Three primary pathways underlie the genetic and epigenetic actions of EDCs: (a) DNA methylation, (b) histone modifications, and (c) altered mRNA expression. During DNA methylation, a methyl group is added to DNA, which can suppress or activate gene expression, resulting in long-term alterations in transcriptional profiles [43].

EDCs can promote histone modifications, altering the proteins around which DNA is wrapped, thereby modulating chromatin structure and influencing gene accessibility for transcription. Additionally, EDCs can induce aberrant expression of microRNAs (miRNAs) without altering the underlying DNA sequence [44].

A notable example is BPA. While some studies indicate that BPA inhibits 11β-HSD1 and 11β-HSD2 in human and rat tissues, others suggest potential increases in mRNA expression and enzymatic activity; for instance, BPA can elevate the mRNA expression levels of various genes involved in lipogenesis regulation [45].

According to the National Health and Nutrition Examination Survey (NHANES), BPA exposure is associated with dental caries, diabetes, and obesity, with the highest urinary BPA levels observed [46]; however, these NHANES datasets are unable to establish causal relationships.

### 6.5. Mechanisms of EDC Action on the Gut Microbiota

EDC exposure has been associated with alterations in gut microbiota composition and function in experimental models and, to a lesser extent, in human studies. These alterations may contribute to microbial dysbiosis, increased intestinal permeability and chronic low-grade inflammation all of which are associated with various pathologies, including those affecting fertility [25].

In addition to these mechanisms, EDCs may also influence immune system function, endocrine signalling, metabolic and oxidative pathways, epigenetic and transcriptional regulation, and the microbiota–xenobiotic metabolism feedback loop.

For example, given the bidirectional relationship between the gut microbiome and sex hormone homeostasis [47] and considering that EDCs are capable of mimicking or blocking endogenous hormones, such interference can indirectly alter microbial ecology.

Although further research is required, there is a strong correlation between EDC exposure and increased intestinal mucosal permeability (“leaky gut”), which may permit bacteria, bacterial toxins, and other substances to enter the bloodstream, thereby inducing systemic inflammation [25]. Leaky gut is also known to be associated with the pathogenesis and progression of gastrointestinal diseases [48].

As described in Chapter 4, oxidative stress, characterized by an imbalance between the generation and elimination of ROS, occurs not only in the inflamed intestinal mucosa but also extends into the deeper layers of the intestinal wall [49], activating redox-sensitive transcription factors that upregulate the expression of pro-inflammatory cytokines and thereby perpetuate the inflammatory cascade [50].

The pathways through which EDCs act on the gut microbiota also involve the aforementioned epigenetic modifications, and over the long term, may result in persistent dysbiosis and altered host–microbiota interactions. The microbiota can transform certain EDCs into metabolites that modify their toxic properties, while other EDCs can alter metabolic enzymes required for the conversion of cholesterol into steroid hormones.

This establishes bidirectional feedback loop between chemical exposure and microbial homeostasis. At the intestinal level, EDCs have been shown to reduce fecal levels of short-chain fatty acids while increasing systemic lipopolysaccharide concentrations and gut permeability [51].

### 6.6. Mechanisms of EDC Action on Brown and Beige Adipose Tissue, Disrupting Thermoregulation

Both synthetic and natural EDCs have the potential to induce adipose tissue dysfunction in white, brown, and beige adipocytes [52]. Brown and beige adipose tissues activate thermogenesis in response to cold exposure by dissipating chemical energy as heat [53]. EDCs can also indirectly impair thermoregulation by disrupting thyroid axis–regulated TH homeostasis [54].

To facilitate interpretation of the heterogeneous evidence base, Table 1 summarizes the major mechanisms discussed in this review, their principal metabolic or clinical implications, and the primary level of supporting evidence.

## 7. Clinical Implications of EDC Exposure in Obesity-Related Metabolic and Endocrine Dysfunction

The clinical consequences of EDC-induced obesity are multifaceted and can be categorized by affected organ systems for clarity, including: metabolic and endocrine; cardiovascular; reproductive; neurological; respiratory; musculoskeletal; and general health.

Experimental evidence indicates that several EDCs may influence body weight and adipose tissue biology by increasing adipocyte number or size, promoting adipogenesis, altering lipid metabolism and affecting metabolic programming. In humans, however, the evidence is mainly associative, and potential transgenerational effects remain plausible but not definitively established. Exposure to obesogenic EDCs in utero and postnatally has been associated with increased adipogenesis and the prevalence of obesity, as these chemicals can alter stem cell differentiation and disrupt the developmental programming of metabolic pathways [17].

Several EDCs have been associated with insulin resistance and pancreatic β-cell dysfunction, particularly in experimental studies. Human observational data also suggest associations between exposure biomarkers and diabetes-related outcomes, but these findings should be interpreted cautiously because they do not establish direct causality [55]. They are also associated with glucose intolerance, as observed in animal studies, and with cardiovascular complications, including hypertension, endothelial dysfunction, and arterial stiffness, which may subsequently exacerbate ischemic heart disease by inducing oxidative stress and systemic inflammation [56], ultimately increasing cardiovascular morbidity and mortality.

This suggests that obesogenic EDCs may also function as diabetogens, a concept reflected in the term “diabesity” introduced in the 1970s to describe the link between diabetes and obesity. The diabetogenic action of certain EDCs may help explain the Metabolically Obese Normal Weight (MONW) phenotype—normal weight individuals with metabolic dysregulation comparable to obese individuals—recognized as a risk factor for cardiovascular disease and type 2 diabetes [57].

Although MONW is not currently diagnosed in routine clinical practice, it is recognized as a risk factor for the development of cardiovascular disease and type 2 diabetes.

This is partly because Body Mass Index (BMI) is an imperfect measure of body weight or adiposity. Another clinical consequence of EDC-induced obesity is non-alcoholic fatty liver disease (NAFLD), with POPs being recognized as having a notable influence on the development, initiation, and progression of NAFLD [58]; however, further studies are required to assess the combined effects of various EDCs on NAFLD pathogenesis.

Since it is known that EDCs can induce thyroid dysfunction, this in turn has a clear association with obesity, which often coexists with thyroid diseases [18], and from this perspective obesity has been associated with hypothyroidism and thyroid nodules [59]. Despite this, the causality between obesity and hypothyroidism is still controversial and need further studies.

The clinical implications of EDC exposure should be interpreted according to the strength of evidence supporting each outcome. The most consistent evidence relates to experimental and mechanistic findings showing that selected EDCs may interfere with adipogenesis, lipid accumulation, insulin signalling, mitochondrial function, oxidative stress and inflammatory activation. In humans, the evidence is mainly observational and supports associations between selected exposure biomarkers and obesity-related phenotypes, insulin resistance, altered lipid metabolism, metabolic syndrome or selected cardiometabolic risk markers. Therefore, these outcomes should be interpreted as biologically plausible and variably supported, rather than as definitively caused by EDC exposure.

By contrast, broader clinical outcomes, including cardiovascular disease, hepatic dysfunction, reproductive disorders, neurological effects, respiratory disease and musculoskeletal consequences, are supported by heterogeneous levels of evidence. Some are linked to EDC exposure through experimental mechanisms or limited observational associations, whereas others remain indirect consequences of obesity-related metabolic dysfunction. Accordingly, broad disease attribution should be avoided, and clinical associations should be interpreted in light of potential confounding, co-exposures, exposure timing, mixture effects, interindividual susceptibility and variability in outcome definitions.

## 8. Prevention and Mitigation Strategies for EDC-Induced Obesity

Addressing obesity linked to EDC exposure requires coordinated efforts at multiple levels, including personal lifestyle modifications, supportive public and community policies, and professional guidance from healthcare providers. As EDCs have become widespread and entered the food chain, individuals may lower their exposure by limiting the intake of processed and canned products and opting instead for fresh, organically produced foods.

Healthcare providers should educate the public, promoting healthy lifestyles from the prenatal period, continuing with the birth period, childhood, and adulthood. Promoting a healthy and balanced lifestyle is fundamental for both the prevention of general obesity and the prevention of EDCs-induced obesity.

In this sense, we can also mention WHO and government actions such as IPCS programme and Registration, Evaluation and Authorisation of Chemicals scheme (REACH) regarding restricting the use of products with high EDC content.

## 9. Discussions

Investigating the health impacts of EDCs presents considerable challenges. In particular, determining their direct contribution to obesity is complex due to the wide range of interacting causes and underlying drivers involved. System-level analyses of obesity have identified more than one hundred interrelated factors, feedback mechanisms, and contributing processes that often operate simultaneously. When narrowing the focus to EDCs—especially those with obesogenic properties—the level of complexity increases even further.

Understanding highly complex phenomena often necessitates a reductionist approach, whereby individual components are studied in the hope of elucidating the functioning of the whole—whether at the level of molecular mechanisms, transmission pathways, or the whole organism as an integrated biological system.

Although our article focused solely on the factors that contribute to the promotion and development of obesity, this does not imply that the group of obesogens less clearly or weakly associated with obesity is less harmful. These substances may exert detrimental effects through other mechanisms and pathways not examined in this analysis.

The strength of evidence differs substantially across the mechanisms discussed in this review. The most consistent support comes from experimental and mechanistic studies showing that selected obesogenic EDCs, particularly organotins and some phthalate metabolites, can interfere with adipocyte differentiation, lipid accumulation and nuclear receptor signalling, including PPARγ/RXR-related pathways. Evidence is also relatively consistent for the ability of several EDCs to induce oxidative stress, mitochondrial dysfunction, inflammatory activation and endocrine interference in experimental models. However, translation of these mechanisms to humans remains more complex. Human studies mainly provide associative evidence linking exposure biomarkers, such as bisphenols, phthalate metabolites or persistent organic pollutants, with obesity-related phenotypes, insulin resistance, altered lipid metabolism or metabolic syndrome. These associations are biologically plausible but may be influenced by confounding factors, differences in exposure measurement, chemical mixtures, timing of exposure and interindividual susceptibility.

Other areas remain more uncertain. Evidence regarding long-term neuroendocrine programming, transgenerational transmission, gut microbiota-mediated effects and brown or beige adipose tissue dysfunction is promising but still incomplete, particularly in humans. Therefore, these mechanisms should be interpreted as plausible and hypothesis-generating rather than definitively causal. This distinction is important because mechanistic plausibility does not necessarily establish human causality. Future studies should combine longitudinal human cohorts, repeated exposure assessment, mixture-based toxicology, mechanistic biomarkers and standardized metabolic outcomes to better clarify which EDC-related pathways have the strongest clinical relevance. Consequently, the precise cumulative impact remains uncertain and cannot be reliably predicted. Furthermore, individual responses to obesogenic EDC exposure vary considerably.Inconsistencies and sources of heterogeneity should also be considered when interpreting the obesogenic relevance of EDCs. Although several experimental and epidemiological studies support associations between selected EDCs and obesity-related phenotypes, the evidence is not uniform across all compounds, populations or outcomes. Some human studies report weak, null or inconsistent associations, particularly when exposure is assessed at a single time point or when outcomes are defined using broad anthropometric measures such as BMI. These discrepancies may reflect differences in exposure assessment, chemical half-life, timing of exposure, population age, sex, genetic susceptibility, baseline metabolic status, diet, physical activity, socioeconomic context and co-exposure to other environmental chemicals.

Experimental findings may also be heterogeneous. Differences in cell type, animal species, developmental stage, exposure route, dose, duration and metabolic background can influence whether an obesogenic phenotype is observed. Moreover, endocrine-disrupting chemicals may exert low-dose or context-dependent effects, while real-world exposure usually occurs as mixtures rather than as isolated compounds. These factors make it difficult to compare studies directly and may partly explain why some findings appear contradictory. Therefore, the evidence should be interpreted as strongest for selected mechanistic pathways and specific compounds, while the overall contribution of EDC exposure to human obesity remains an evolving field requiring longitudinal cohorts, repeated exposure measurements, mixture-based models and standardized metabolic outcomes.

## 10. Limitations

This review has several methodological limitations that should be acknowledged. Although the literature search was structured, this manuscript was designed as a narrative review and was not conducted according to a full PRISMA framework. Therefore, no PRISMA flow diagram, formal risk-of-bias assessment, or quantitative grading of evidence certainty was performed. This represents a methodological limitation, particularly given the heterogeneity of the available evidence, which includes in vitro studies, animal models, observational human studies, systematic reviews, and regulatory reports. Consequently, the conclusions should be interpreted as a narrative synthesis of the current evidence rather than as a systematic or quantitatively graded assessment. A further limitation is that several mechanistic pathways discussed in this review are derived mainly from in vitro and animal studies. While these models are valuable for identifying receptor-level, cellular and molecular mechanisms, they do not necessarily demonstrate that the entire pathway operates in the same manner in humans. This is particularly relevant for neuroendocrine programming and oxidative stress–inflammatory cascades, where individual components are biologically plausible and experimentally supported, but the full causal sequence linking EDC exposure to human obesity remains incompletely established.

## 11. Conclusions

Obesogens represent plausible environmental contributors to obesity and metabolic dysfunction, supported by strong mechanistic evidence for selected compounds and by associative human data for several exposure biomarkers. However, the strength of evidence varies across chemical classes, outcomes and mechanisms, and direct human causality remains difficult to establish for many EDC-related effects. Reducing exposure to well-characterized obesogenic EDCs remains a relevant precautionary and public health objective. The mechanisms of action described have typically been analyzed as if they operated independently, for each class of EDCs separately.

In reality, exposure to EDCs is often multiple, with overlapping pathways and mechanisms of action that produce effects that remain insufficiently understood. The human body functions as an integrated whole, yet it is so complex that it has traditionally been studied through discrete anatomical structures. The same applies to endocrine disruptors.

For these reasons, exposure reduction, continued mechanistic and longitudinal human research, mixture-based toxicological assessment and public education are essential. Future studies should clearly distinguish experimental plausibility from human causal inference in order to better define the clinical relevance of obesogenic EDC exposure. However, the available evidence is heterogeneous, and null or inconsistent findings have been reported, particularly in human observational studies. This variability highlights the need for cautious interpretation and for future studies using repeated exposure assessment, longitudinal design, mixture-based approaches and standardized obesity-related outcomes.

## Figures and Tables

**Table 1:** Major obesogenic EDC-related mechanisms, associated outcomes, and primary levels of evidence.

Mechanism or Pathway	Main Biological Effects	Associated Metabolic or Clinical Outcomes	Primary Level of Evidence	Current Interpretation
PPARγ/RXR activation and adipogenesis	Adipocyte differentiation, lipid storage, adipose tissue expansion	Increased adiposity, altered lipid metabolism, insulin resistance	In vitro; animal; limited human observational evidence	Relatively well supported experimentally; direct human causality remains unconfirmed
Estrogen receptor-dependent and independent signalling	Altered hormonal signalling, adipocyte function and adipokine regulation	Adiposity, metabolic dysfunction, reproductive effects	In vitro; animal; human observational evidence	Biologically plausible, with heterogeneous evidence across compounds and tissues
Mitochondrial dysfunction and oxidative stress	Increased ROS production, impaired oxidative phosphorylation and cellular stress	Insulin resistance, metabolic dysfunction, inflammatory activation	In vitro; animal; human biomarker associations	Strong experimental support; partial and associative confirmation in humans
Pro-inflammatory activation	NF-κB activation, cytokine and chemokine production, macrophage recruitment	Chronic low-grade inflammation, insulin resistance, cardiometabolic risk	In vitro; animal; human observational evidence	Mechanistically supported; clinical relevance remains heterogeneous
Neuroendocrine programming	Altered hypothalamic appetite and satiety signalling, leptin and insulin signalling	Altered food intake, energy expenditure and body weight regulation	Mainly animal and mechanistic evidence; limited human observational evidence	Emerging mechanism that remains incompletely established in humans
Genetic and epigenetic regulation	DNA methylation, histone modification and altered non-coding RNA expression	Developmental metabolic programming and possible intergenerational effects	In vitro; animal; limited human observational evidence	Biologically plausible; long-term human relevance remains uncertain
Gut–brain–microbiota interactions	Dysbiosis, altered intestinal permeability, microbial metabolite changes and inflammatory signalling	Obesity-related metabolic dysfunction and insulin resistance	Mainly animal and mechanistic evidence; limited human evidence	Emerging mechanism requiring further human validation
Brown and beige adipose tissue dysfunction	Impaired thermogenesis and reduced energy expenditure	Increased susceptibility to adiposity and metabolic dysfunction	Mainly in vitro and animal evidence	Incompletely established in humans
Thyroid hormone disruption	Altered thyroid hormone synthesis, transport, metabolism or action	Reduced energy expenditure and altered metabolic regulation	Experimental and human observational evidence	Plausible association; causality and clinical magnitude remain uncertain
EDC mixture and cumulative exposure effects	Additive, synergistic or antagonistic effects across shared pathways	Obesity-related phenotypes, metabolic syndrome and cardiometabolic risk	Experimental mixture studies; human observational studies; systematic reviews	Highly relevant to real-world exposure but methodologically challenging

## Data Availability

No new data were created or analyzed in this study.

## Abbreviations

The following abbreviations are used in this manuscript:

11β-HSD1	11β-hydroxysteroid dehydrogenase type 1
11β-HSD2	11β-hydroxysteroid dehydrogenase type 2
AgRP	Agouti-related peptide
ARC	Arcuate nucleus
BMI	Body mass index
BMP	Bone morphogenetic protein
BPA	Bisphenol A
BPAF	Bisphenol AF
BPC	Bisphenol C
CART	Cocaine- and amphetamine-regulated transcript
CCL2	Chemokine C-C motif ligand 2
CCL5	Chemokine C-C motif ligand 5
DAMPs	Damage-associated molecular patterns
DDE	1,1-dichloro-2,2-bis(p-chlorophenyl)ethylene
DDT	Dichlorodiphenyltrichloroethane
DEHP	Di-2-ethylhexyl phthalate
DNA	Deoxyribonucleic acid
EDCs	Endocrine-disrupting chemicals
ER	Estrogen receptor
ETC	Electron transport chain
FSH	Follicle-stimulating hormone
HPA axis	Hypothalamic–pituitary–adrenal axis
IPCS	International Programme on Chemical Safety
IR	Insulin resistance
LH	Luteinizing hormone
MEHP	Mono-(2-ethylhexyl) phthalate
miRNAs	microRNAs
MONW	Metabolically obese normal weight
mRNA	Messenger RNA
MSCs	Mesenchymal stromal cells
NAFLD	Non-alcoholic fatty liver disease
NF-κB	Nuclear factor kappa B
NHANES	National Health and Nutrition Examination Survey
NLRP3	NOD-like receptor family pyrin domain containing 3
NPY	Neuropeptide Y
Nrf2	Nuclear factor erythroid 2-related factor 2
PCBs	Polychlorinated biphenyls
PHBPs	Polyhalogenated bisphenols
POMC	Pro-opiomelanocortin
POPs	Persistent organic pollutants
PPARα	Peroxisome proliferator-activated receptor alpha
PPARγ	Peroxisome proliferator-activated receptor gamma
PVC	Polyvinyl chloride
REACH	Registration, Evaluation, Authorisation and Restriction of Chemicals
ROS	Reactive oxygen species
RXR	Retinoid X receptor
TBT	Tributyltin
TH	Thyroid hormone
UPR	Unfolded protein response
WHO	World Health Organization
